# Magnitude of husbands’ involvement in skilled birth attendant service utilization in Deder town, Oromiya, Ethiopia

**DOI:** 10.1186/s12884-022-05181-1

**Published:** 2022-11-15

**Authors:** Abdusamed Mohammed, Gizaw Kifle, Furo Beshir, Abas Mume, Yasin Abdullahi, Remzia Abdulwehab

**Affiliations:** 1 Department of Midwifery, Harar Health Sciences College, Harar, Ethiopia; 2 Department of Medical Laboratory Sciences, Harar Health Science College, Harar, Ethiopia; 3 Department of Nursing, Harar Health Sciences College, Harar, Ethiopia; 4 Department of Business and Economics, Harar Health Science College, Harar, Ethiopia; 5 Department of Public Health, Harar Health Science College, Harar, Ethiopia

**Keywords:** Delivery, Husbands’ involvement, Skilled birth attendants

## Abstract

**Background:**

Husbands’ involvement strategies are employed to support their wives to access care, address the influence of gender inequality on maternal and newborn health, and promote husbands’ positive involvement as fathers. Yet, evidence of the husbands’ involvement in skilled birth attendant service utilization in Ethiopia is scarce, which limits the facility to improve institutional delivery services. Therefore, this study aimed to assess the magnitude of husbands’ involvement in skilled birth attendant service utilization among fathers of children of less than one year in Deder town, Oromiya, Ethiopia.

**Methods:**

A community-based cross-sectional study was conducted using a structured questionnaire among 399 fathers selected by a simple random sampling technique. Data were collected through face-to-face interview from March 24 to April 20, 2021. Data entry was done by Epi-data version-4.2 and analyzed with SPSS version-21. Descriptive statistics used frequency, mean and median and presented by tables and figures. The level of significance was set at *p*-value ≤0.05 for multivariable logistic regression and an adjusted odds ratio with a 95% confidence interval was used to measure the degree of statistical significance of dependent and independent variables.

**Result:**

The prevalence of husbands’ involvement in skilled birth attendant service utilization was 84.2%. The age group of 25 to 29 years (AOR = 2.63, CI: 1.124–6.142), prior knowledge of skilled-delivery attendants (AOR = 2.75, CI: 1.456–5.205) and good attitude towards skilled birth attendants (AOR =2.46, CI: 1.227–4.948) were statistically significant determinants of husbands’ involvement in skilled birth attendants service utilization.

**Conclusion and recommendation:**

This study revealed that the husbands’ involvement in skilled birth attendants was high. Age, level of knowledge and attitude were the significant determinants of husbands’ involvement in promoting skilled-delivery attendants. Therefore, improving husbands’ level of knowledge and attitude towards skilled birth attendants is needed.

## Background

Pregnancy and childbirth is natural and often an eventful process. All mothers are at risk of developing complications at any time throughout their pregnancy and childbirth period. A vast majority of maternal deaths are due to preventable direct obstetric causes, such as hemorrhage, infection, obstructed labor, unsafe abortion and high blood pressure [1]. These causes can be detected and managed early during antenatal care (ANC) and the intrapartum period by existing and well-known medical interventions.

Delay to decide to seek care is one of the contributing factors to maternal morbidity and mortality and is preventable [2]. Hence, access to skilled health services during pregnancy, childbirth and postnatal period for obstetric care are one of the strongest determinants of maternal and newborn health outcomes [3]. Unfortunately, the percentage of women in Ethiopia using skill birth attendance (SBA) has been very low, according to the 2016 Ethiopian Demographic Health Survey (EDHS) [4].

A Prompt decision in seeking reproductive health services like having a skilled birth attendant (SBA) at every delivery is markedly influenced by husbands [5, 6]. Husbands typically serve as gatekeepers of women’s reproductive health including decisions about where they will be delivering as different evidence shows [5, 7].

In African countries, men generally are considered to be the decision-makers regarding the location at which their spouse should give birth [7, 8]. Therefore, male involvement in maternal health care services is a building block for ensuring women’s and children’s wellbeing.

Maternal mortality is a key indicator of international development and its reduction has long been and continues to be a global challenge, particularly in low-income countries including Ethiopia. Globally, in 2015, an estimated 303,000 women died as a result of pregnancy and childbirth-related complications [9, 10].

Almost 99% (302, 000) of global maternal deaths occurred in developing countries, with the maternal mortality ratio (MMR) of 239 per 100,000 live births which are more than 14 times higher compared to the developed regions (12 maternal deaths per 100,000 live births). Sub-Saharan Africa (SSA) countries alone accounted for 66% of maternal deaths with maternal mortality ratio of 546 per 100,000 live births (201,000), followed by Southern Asia 180 per 100,000 live births (66000) [9].

In Ethiopia, the maternal mortality ratio is estimated at 353 per 100,000 live births according to World Health Statistics 2018 [10], indicating a significant improvement from that reported in 2011 EDHS 676/100,000 live births. However, this figure is far from the millennium development goal (MDG) target of 267 maternal deaths per 100,000 live births by 2015 [9].

Lack of maternal health services has been considered one of the factors that resulted in the slow progress of maternal mortality reduction programs. However, in some cases where the different services exist, husbands are reported to forbid their wives from seeking any maternal health care like delivery [11].

Globally, the proportion of deliveries attended by skilled health personnel increased from 59% in 1990 to 71% in 2014 [12]. Yet this leaves more than one in four babies and their mothers without access to crucial medical care during childbirth. In Sub-Saharan African countries, 42% of children birth are assisted by skilled birth attendants while in Ethiopia, it accounts for only 26% as reported by EDHS 2016 [4].

Some studies conducted in different countries indicate that social, cultural, and religious factors play a great role in skilled birth attendant service uptake. Age, harmful traditional practices, the low social status of women, limited female involvement in decision making, family members’ influence and decisions, and women’s limited influence over their families are key factors in SBA service uptake [13, 14]. In addition, religious reasons, the poor attitude of health workers, and the poor quality of care are related to low service uptake [15]. World Health Organization had done different interventions for husbands to increase their involvement in order to tackling the above mentioned problems by increasing the male partners’ involvement in maternal and child health including skill birth attendance [16].

Even if different interventions have done regarding husbands’ involvement to increase their involvement, the evidence of study results in northern Nigeria and Farta district, Northwest Ethiopia revealed that more than 25 and 8.3% of mothers respectively deliver at home due to the influence of their husband not to go to health facility at time of labor [11, 17]. From their studies findings, they recommend that further research be undertaken to determine the prevalence of male partner involvement in promoting skilled birth attendants and associated factors.

The current prevailing literature on the issue did not fully address the prevalence of the problem and factors that are associated with it separately rather than male partners’ involvement in maternal and child health as a whole. And also most researchers’ focus has always been on rural areas regarding maternal and child health-related research due to the intention of high mortality and morbidity at rural areas, while the prevalence and associated factors of male involvement in urban areas gained little attention. Therefore, this study is very curial and timely to identify the magnitude of husbands’ involvement in skilled birth attendant service utilization in the study area.

## Methods and materials

### Study area

The study was conducted in Deder town, Eastern Hararghe zone, Oromiya, Ethiopia from March 24 to April 20, 2021. Deder is located on latitude 9.25° or 9° 15′ north, at 131.8 km from Harar, the capital of the eastern Hararghe zone, and 440.4 km from Addis Ababa, the capital city of Ethiopia. The total population of the town is estimated to be 34,812 out of whom 16,000 were husbands.

### Study design

Community-based cross-sectional study design was employed.

#### Sample size determination, techniques and procedures

The sample size required for this study was calculated based on a single population proportions formula as follows.

n = $\frac{{\left( Z\alpha /2\right)}^2P\left(1-P\right)}{d^2}$ where: *n* = the desired sample required, Zα/2 = the standard normal variable at 95% confidence level = 1.96, *P* = is the prevalence of male partners’ involvement in institutional delivery of spouse 38.2% (taken from a study done at Lemo woreda, Southern Ethiopia 2015) (27) and d = is the margin of error assumed to be tolerated (5%). By considering 10% non-response rate, the final sample size was became 399. Simple random sampling technique was employed to select the study participants.

#### Source and study population

All fathers of less than 1 year of age children in the town were the source population whereas randomly selected fathers of less than 1 year of age children were the study population.

#### Data collection instruments and techniques

Data were collected through face-to-face interviews using the Afan Oromo version structured questionnaire that was originally prepared in English from different kinds of literature [14, 18, 19]. The data collection was conducted by four trained Health Extension Workers and supervised by two BSc Midwives.

#### Operational definitions

***Husband involvement:*** a husband is a male partner in a legal marriage. He is legally married to a woman and thereby has specific rights and duties given by law [19]. Husband involvement in promoting skilled-birth attendants is measured by five questions. Participants who answered ‘***Yes***’ to three or more questions were considered as involved or vice versa.

Knowledge of husband’s involvement in the decision of place of delivery: - five knowledge-related questions was included in the assessment tools to differentiate participants with good and poor knowledge.

- Good knowledge:- Those participants who scored more than or equal to the median value of knowledge related questions
- Poor knowledge:- Those participants who scored less than the median value of knowledge-related questions.

Attitude: -The respondents were asked to reflect on their opinion about skilled-delivery attendants.

- Good attitude: participants who scored more than or equal to the median value of attitudinal related questions.
- Poor attitude: participants who scored less than the median value of attitudinal-related questions.

#### Data processing and analyses

After data collection, data were coded and entered into Epi--data version-4.2. The analysis was made with SPSS version-21. First descriptive analyses like frequency, mean and median were done and presented by tables and figures. Then Hosmer-Lemeshow and Collinearity test were done to check model fitness and the relationship among independent variables respectively. To check for the internal consistency the instrument, the reliability analysis was done and the overall Cronbach’s alpha value was 0.87, reflecting a very high consistency of the instrument.

Finally, binary and multivariable logistic regressions were run to assess the association between the dependent and independent variables. Again, *p*-value ≤0.2 and ≤ 0.05 in binary and multivariable logistic regression respectively were considered significant at a 95% confidence level.

## Results

### Socio-demographic characteristics

All 399 study participants completed the questionnaires making a response rate of 100%. One hundred twenty six (31.6%) were in the age group of 30–34 years. The mean age of the participant was 31.89 ± 5.15 SD years. The study participants were predominantly Oromo [375 (94.0%)] and Muslim [361 (90.5%)] by their ethnicity and religion respectively. One hundred twenty five (31.3%) of the participants were merchants and 178 (44.6%) have completed college and above (Table 1).

**Table 1:** Socio-demographic characteristics of husbands’ involvement in promoting skilled-birth attendants, Deder town, Oromiya, Ethiopia, 2021, (*n* = 399)

Variables	Frequency	Percentage (%)
**Age**		
20–24 yrs.	28	7.0
25–29 yrs.	124	31.1
30–34 yrs.	126	31.6
>/=35 yrs.	121	30.3
**Religion**		
Muslim	361	90.5
Orthodox	22	5.5
Protestant	16	4.0
**Ethnicity**		
Oromo	375	94.0
Amhara	9	2.3
Harari	3	0.7
Gurage	12	3.0
**Occupation**		
Merchants	125	31.3
Private employee	60	15.0
Farmer	49	12.3
Government employee	116	29.1
Labor work and others	49	12.3
**Educational status**		
Read and write only	46	11.5
Primary education (grade 1-8)	57	14.3
Secondary education (grade 9-12)	118	29.6
College and above	178	44.6

### Perceived barriers of husbands’ involvement in skilled birth attendant service utilization

The result of this study showed that more than half of the participants [228(57.1%)] reach the health facility by walking within 30 minutes and below. Regarding cultural related barriers to husbands’ involvement in skilled birth attendant service utilization, about 223 (55.9%) said that child-birth is a woman’s affair that does not require husband participation and 40(10%) believed that discussions with the wife about the place of childbirth were not their culture (Table 2).

**Table 2:** Perceived barriers of husbands’ involvement in skilled birth attendant service utilization, Deder town, Oromiya, Ethiopia, 2021, (*n* = 399)

Variables	Categories	Frequency	Percentage
Time took on walking to reach the nearest health facilities	</=30 minutes	228	57.1
	> 30 minutes	171	42.9
Cost of the health service	Free	307	76.9
	Partially free	92	23.1
Health facilities are open day and night	yes	238	59.6
	No	161	40.4
Childbirth is a woman’s affairs and does not require men’s participation	Yes	223	55.9
	No	176	44.1
Childbirth is natural phenomenon that should not be given much attention	Yes	393	98.5
	No	6	1.5
Discussion with wife about the place of delivery not our culture	Yes	40	10.0
	No	359	90.0
Spouses living together	Yes	364	91.2
	No	35	8.8

### Knowledge of husbands toward skilled-delivery attendance of their wives

All participants reported they had known their spouses had received ANC follow-up but only 202(50.6%) know correctly the recommended minimum number of times that pregnant needs to attend ANC (Table 3).

**Table 3:** Knowledge of husbands toward skilled-delivery attendants, Deder town, Oromiya, Ethiopia, 2021. (*n* = 399)

Variables	Categories	Frequency	Percentage
Mentioned correctly the recommended minimum number of times that pregnant needs to attend ANC	Yes	202	50.6
	No	197	49.4
Institutional delivery has access to skilled attendance	Yes	371	93.0
	No	28	7.0
Institutional delivery prevents delay in getting medical care in a case of emergency	Yes	299	74.9
	No	100	25.1
Institutional delivery has important to get immediate treatment for the mother and newborn	Yes	303	75.9
	No	96	24.1

The result of this study showed that the majority, 295(73.9%), of the participants were found to have good knowledge regarding skilled-delivery attendance.

### Husbands’ attitude to skilled-delivery attendance

The study result showed that 209(52.4%) of husbands had a good attitude towards skilled-delivery attendance, whereas 190 (47.6%) had a poor attitude.

### Husbands’ involvement in skilled birth attendant service utilization

**Fig. 1:**
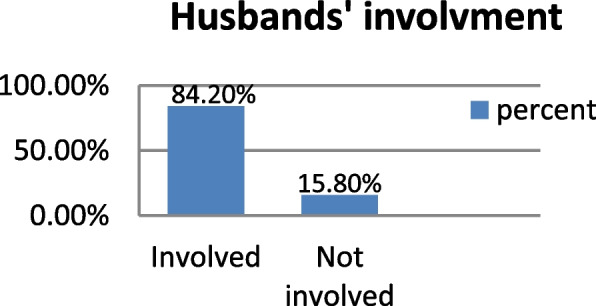
Husbands’ involvement skilled birth attendant service utilization, Deder town, Oromiya, Ethiopia, 2021 (*n* = 39)

The overall husband involvement in skilled birth attendant service utilization was 366(84.2%) (Fig. 1).

Over three fourth (79.4%) of participants have ever gone to health facilities with their wives for antenatal care services in their last pregnancy. About 396 (99.2%) of participants were involved in birth preparedness of a recent child, among whom 362 (90.7%) saved money, 349 (87.5%) prepared essential items for delivery and 197 (49.4%) planned for the place of delivery. Regarding the motives to discuss with health professionals, 77 (19.3%), 139 (34.8%) and 145 (36.3%) have previous experience, listing mass media and personal interest, respectively, as the sources of their motives to be involved in promoting skilled-delivery attendants (Table 4).

**Table 4:** Distribution of husbands’ involvement skilled birth attendant service utilization, Deder town, Oromiya, Ethiopia, 2021, (*n* = 399)

Variable	Categories	Frequency	Percentage
Accompanied their spouse for antenatal care follow-up	Yes	317	79.4
	No	82	20.6
Birth preparedness support by husbands for recent child	Yes	396	99.2
	No	3	0.8
Discussed with health professionals	Yes	165	41.4
	No	234	58.6
Discussed with their friends	Yes	176	44.1
	No	223	55.9
Discussed with their relatives	Yes	328	82.2
	No	71	17.8

### Factors associated with husbands’ involvement in skilled birth attendant service utilization

Results of the bi-variable logistic regression showed that the husbands’ age, educational status, occupational status, knowledge, attitude and educational status were statistically significant determinants of husbands’ involvement in promoting skilled-delivery attendants. But after adjusting the confounding variables, husbands’ age, knowledge and attitude were found to be statistically significant determinants of husbands’ involvement in promoting skill-delivery attendants at an alpha value of 5%.

Husbands who were in the age group of 25 to 29 years old were 2.75 times higher odds of promoting skilled-delivery attendance when compared to those who were in the age group of 35 years and above (AOR = 2.75, CI: 1.169–6.448). Husbands who had good knowledge were 2.83 times higher in promoting skilled-delivery attendants than those who had a poor knowledge (AOR = 2.83, CI: 1.491–5.384).

The odds of promoting skilled-delivery attendant were two times higher among husbands who had good attitude when compared to their counterparts (AOR =2.40, CI: 1.194, 4.842) (Table 5).

**Table 5:** Bivariate and multivariable logistic regression analysis of determinants of husbands’ involvement in skilled-delivery attendants, Deder town, Oromiya, Ethiopia, 2021, (*n* = 399)

Variables	Male involvement		COR(95%CI)	AOR(95%CI)
	Involved	Not Involved		
**Age categories**				
20–24 yrs.	25(89.3%)	3(10.7%)	2.75(.774, 9.750)	3.16(.723,13.816)
25–29 yrs.	111(89.5%)	13(10.5%)	2.82(1.388, 5.710)	2.75(1.169,6.448)^a^
30–34 yrs.	109(86.5%)	17(13.5%)	2.11(1.096, 4.077)	1.82(.838,3.954)
>/=35 yrs.	91(75.2%)	30(24.8%)	1	1
**Educational status**				
Read and write only	29(63.0%)	17(37.0%)	.146(0.065,0.327)	3.53(.245,50.853)
Primary education [1–8]	41(71.9%)	16(28.1%)	.23(0.099, 0.484)	1.70(.140,20.733)
Secondary education [9–12]	102(86.4%)	16(13.6%)	.54(0.255,1.162)	1.734(.193,15.610)
College and above	164(92.1%)	14(7.9%)	1	1
**Occupational status**				
Merchant	118(94.4%)	7(5.6%)	6.09(2.258,16.411	6.29(.473,83.747)
Private employee	53(88.3%)	7(11.7%)	2.73(0.994,7.519)	2.51(.223,28.101)
Farmer	29(59.2%)	20(40.8%)	.52(.223,1.228)	.52(.180,1.505)
Governmental employee	100(86.2%)	16(13.8%)	2.26(0.989,5.150)	1.38(.363,5.217)
Labor work and others	36(73.5%)	13(26.5%)	1	1
**Level of knowledge about skilled-delivery attendant**				
Good knowledge	266(90.2%)	29(9.8%)	4.46(2.542, 7.808)	2.83(1.491,5.384)^a^
Poor knowledge	70(67.3%)	34(32.7%)	1	1
**Attitude toward the skilled-delivery attendant**				
Good attitude	194(92.8%)	15(7.2%)	4.37(2.354, 8.118)	2.40(1.194,4.842)^a^
Poor attitude	142(74.7%)	48(25.3%)	1	1

^a^ Show statistically significant association in multivariate logistic regression at *p*-value ≤0.05

## Discussion

This study was conducted to assess the magnitude of male involvement and associated factors in Deder, Oromia, Ethiopia. The current study found that the prevalence of husbands’ involvement in skilled birth attendant service utilization was 84.2%. This finding is higher than the study done in Mareka woreda (41.3%), southeast Ethiopia and Lemmo woreda (38.2%), southern Ethiopia respectively [13, 18]. The finding is relatively in line with findings of studies done on husbands’ involvement in skilled birth attendant service utilization in Kenya (68%), Ghana (73.3%) and Myanmar (69.7%) [20–22]. These big levels of variation of male involvement might be occurred due to the variation of the study area, because those two previous studies were done at rural level whereas, the present study was done in a relatively urban area.

Regarding the determinants of husbands’ involvement in skilled birth attendant service utilization, this study revealed that the age of the husbands was statistically significant in promoting skilled delivery attendance. This finding was similar to studies conducted in Mareka Woreda, Ethiopia and Nigeria [8, 13]. This might be because younger men are more heroic and likely to challenge cultural norms. Additionally, they might have a better chance of an education which is known to positively influence health-seeking behaviors in this age group most of the time.

However, the age category which had an association with husbands’ involvement in this study was older when compared to other studies done in Ghana relatively [22]. The difference could be due to different educational statuses, socio-cultural characteristics of the study participants and health care strategies of the country regarding husbands’ involvement in maternal & child health.

Knowledge about skilled-delivery attendance was another statistically significant determinant for husbands’ involvement in skilled birth attendant service utilization. This finding was consistent with study done in Myanmar and Lemmo woreda, Southern Ethiopia [18, 23] in which having good knowledge about skilled delivery could influence husbands’ involvement in skilled birth attendant service utilization. The reason for this might be explained by the possibility that those with good knowledge understand well possible birth complications, benefits, advantages and disadvantages; so they encourage their spouses to gate skill delivery attendance.

Husbands’ attitude was also another statistically significant determinant for husbands’ involvement in skilled birth attendant service utilization. This finding was similar to other studies done in Mareka woreda, southeast Ethiopia, Kenya and Myanmar respectively [14, 21, 23]. The possible explanation for this similarity is that getting new and healthy child is the desire of all husbands; so they encourage their spouses to gate skill delivery attendance.

## Conclusion and recommendations

This study revealed that husbands’ involvement in skilled-delivery attendance was high. Husbands’ age-related experiences, level of knowledge and having a positive attitude were the contributing factors to husbands’ involvement in promoting skill delivery attendants. Therefore, awareness creation about institutional delivery to help the husbands know about skilled-attendance institutional delivery and creating a community culture that acts on creating a positive attitude towards skilled-attendance institutional delivery is recommended.

### Limitations of the study

Since this study interviewed the husbands directly, there may be social desirability bias. Another limitation of this study was that study focuses on urban area only due to budget constraints and hence we would like to recommend another study to be conducted by incorporating the study participants from rural area and/or mixed method study.

## Data Availability

All necessary materials of this manuscript will be obtained from the corresponding author upon the request.
